# Correction: Hung et al. Anti-Inflammatory Fibronectin-AgNP for Regulation of Biological Performance and Endothelial Differentiation Ability of Mesenchymal Stem Cells. *Int. J. Mol. Sci.* 2021, *22*, 9262

**DOI:** 10.3390/ijms232314998

**Published:** 2022-11-30

**Authors:** Huey-Shan Hung, Kai-Bo Chang, Cheng-Ming Tang, Tian-Ren Ku, Mei-Lang Kung, Alex Yang-Hao Yu, Chiung-Chyi Shen, Yi-Chin Yang, Hsien-Hsu Hsieh, Shan-hui Hsu

**Affiliations:** 1 Graduate Institute of Biomedical Science, China Medical University, Taichung 40402, Taiwan; 2 Translational Medicine Research, China Medical University Hospital, Taichung 40402, Taiwan; 3 College of Oral Medicine, Chung Shan Medical University, Taichung 55015, Taiwan; 4 Department of Medical Education and Research, Kaohsiung Veterans General Hospital, Kaohsiung 43302, Taiwan; 5 Ministry of Health & Welfare, Changhua Hospital, Changhua 51341, Taiwan; 6 Neurological Institute Head of Department of Neurosurgery, Taichung Veterans General Hospital, Taichung 407204, Taiwan; 7 Department of Physical Therapy, Hung Kuang University, Taichung 433304, Taiwan; 8 Basic Medical Education Center, Central Taiwan University of Science and Technology, Taichung 406053, Taiwan; 9 Blood Bank, Taichung Veterans General Hospital, Taichung 407204, Taiwan; 10 Institute of Polymer Science and Engineering, National Taiwan University, Taipei 10617, Taiwan

The authors wish to make the following correction to this paper [1]:

In the original publication, there was a mistake in Figure 1 as published. In Figure 1C, the original AFM images showed the same between FN group and FN-AgNP (15.1) group. The image of FN group in Figure 1C was misplaced. However, the results of Ra and Rq value in Figure 1D demonstrated the difference between FN group and FN-AgNP (15.1) group, which should be correct; the data were obtained from different groups instead of the same. Next, there was a mistake in Figure 7 as published. In Figure 7A, the original images of immunohistochemistry staining for endothelialization marker CD31 showed the same between FN group and FN-AgNP (15.1) group. The image of FN group in Figure 7A was misplaced. However, the semi-quantified result based on CD31 expression intensity in Figure 7B were demonstrated as different between the FN group and the FN-AgNP (15.1) group; the results should be correct and be obtained from different samples instead of the same. The correct figures appear below.

The authors state that the scientific conclusions are unaffected. This correction was approved by the Academic Editor. The original publication has also been updated.

## Figures and Tables

**Figure 1:**
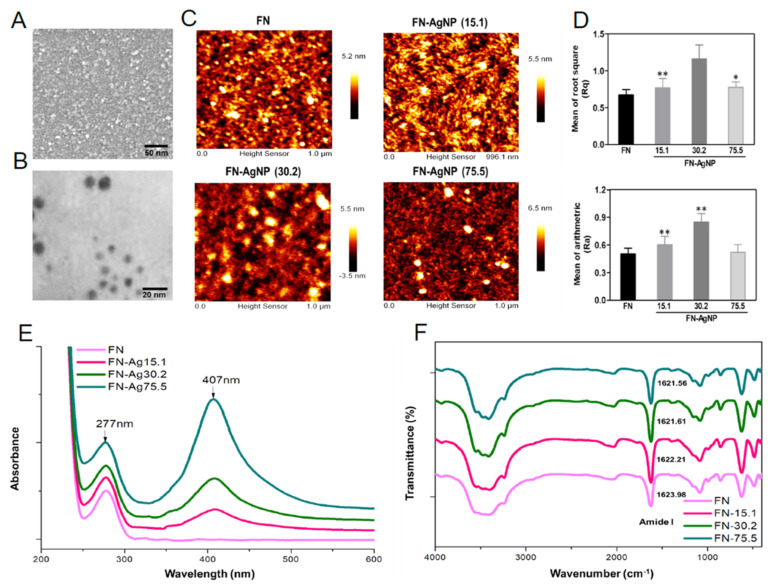
Materials characterization. The preparation procedure of fibronectin-silver nanoparticle (FN-Ag) composites. (**A**) SEM images of AgNP and (**B**) TEM image of the diameter of AgNP was about 5 nm. (**C**) Preparation of FN-AgNP nanocomposites. The surface topography of pure FN and FN-AgNP containing various amount of Ag were observed by AFM. (**D**) Rq is the roughness, while Ra represents as the average roughness of each material. * *p* < 0.05, ** *p* < 0.01: greater than the control (FN). (**E**) UV-Visible absorption peak of pure FN and FN-Ag containing various amount of AgNP (~15.1 ppm, ~30.2 ppm and ~75.5 ppm). (**F**) FTIR spectrum of pure FN and FN-Ag nanocomposites in the total wave number 400 cm^−1^ to 4000 cm^−1^. Data results from one representative experiment of three independent experiments.

**Figure 7:**
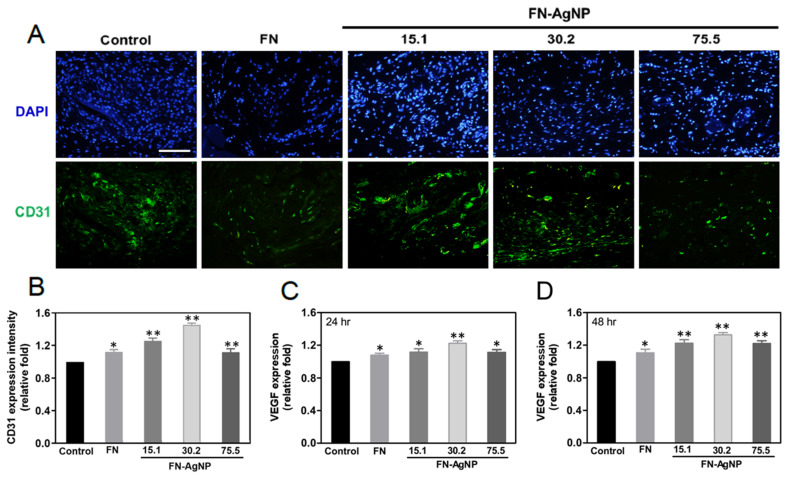
Immunohistochemistry staining for endothelialization marker CD31 affected by the implant materials. (**A**) The histology images for CD31 were showed after implanting various materials for 4 weeks. Scale bar = 100 μm. (**B**) The CD31 expression was also investigated and then semi-quantified according to fluorescence intensity. * *p* < 0.05; ** *p* < 0.01: higher than the control (*n* = 5). (**C**,**D**) The expression level of VEGF protein was evaluated via ELISA assay at 24 and 48 h, then the fluorescence intensity were semi-quantified by Image J software. Data are the mean ± SD. * *p* < 0.05; ** *p* < 0.01: greater than the control (*n* = 5).

## References

[1] Hung H.-S., Chang K.-B., Tang C.-M., Ku T.-R., Kung M.-L., Yu A.Y.-H., Shen C.-C., Yang Y.-C., Hsieh H.-H., Hsu S.-h. (2021). Anti-Inflammatory Fibronectin-AgNP for Regulation of Biological Performance and Endothelial Differentiation Ability of Mesenchymal Stem Cells. Int. J. Mol. Sci..

