# Supplementary material for: Anti-Inflammatory Fibronectin-AgNP for Regulation of Biological Performance and Endothelial Differentiation Ability of Mesenchymal Stem Cells
Source: Int J Mol Sci. 2021 Aug 26;22(17):9262. doi: 10.3390/ijms22179262 (PMC8430779; doi:10.3390/ijms22179262)
Supplement: Supplementary file 1 [file ijms-22-09262-s001.zip › ijms-1324192-supplementary.pdf]

Figure S1

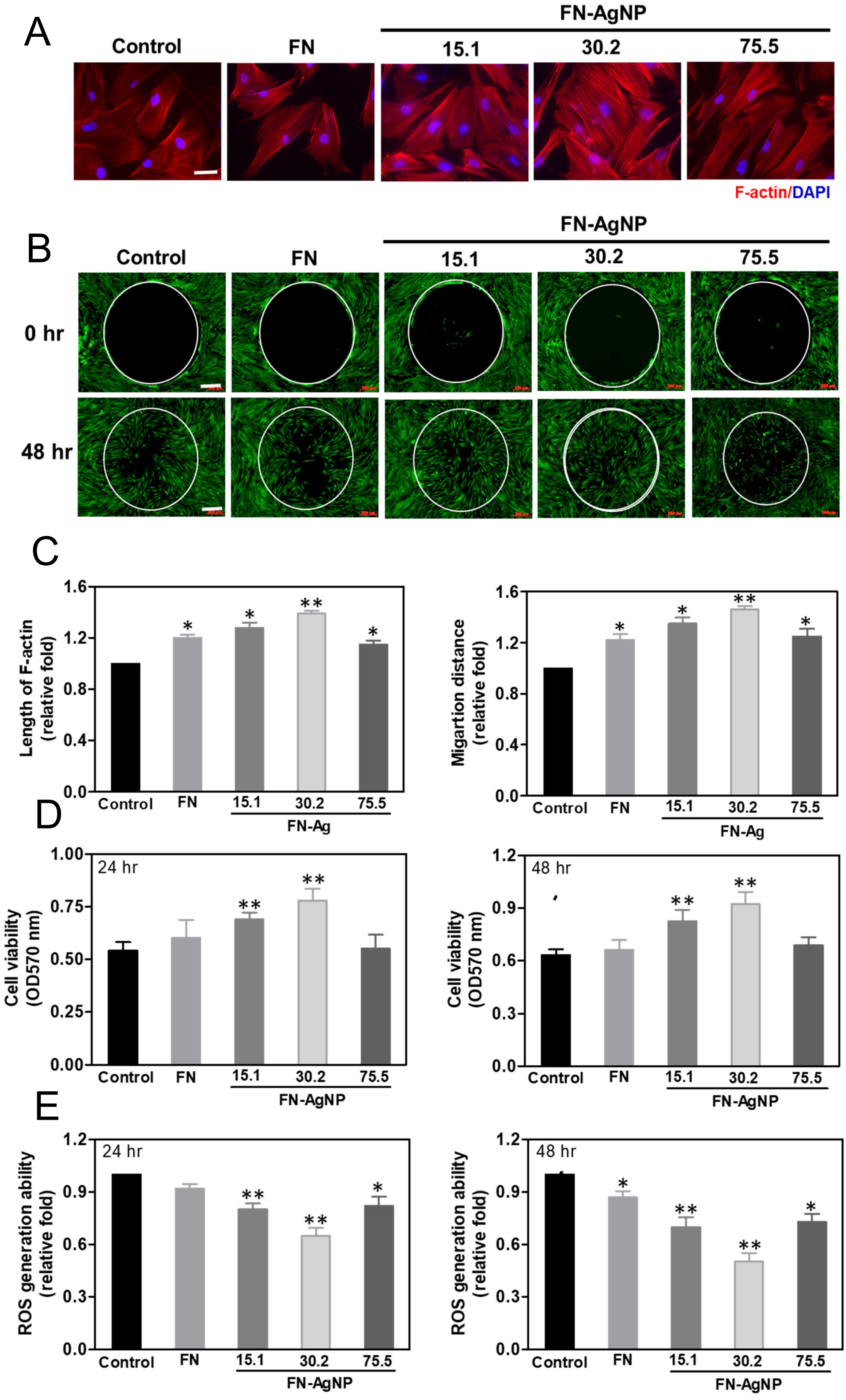

A
Control
FN
FN-AgNP
15.1
30.2
75.5
F-actin/DAPI
B
Control
FN
FN-AgNP
15.1
30.2
75.5
0 hr
48 hr
C
Length of F-actin (relative fold)
1.6
1.2
0.8
0.4
0.0
Control
FN
15.1
30.2
75.5
FN-Ag
Migartion distance (relative fold)
1.6
1.2
0.8
0.4
0.0
Control
FN
15.1
30.2
75.5
FN-Ag
D
24 hr
Cell viability (OD570 nm)
1.00
0.75
0.50
0.25
0.00
Control
FN
15.1
30.2
75.5
FN-AgNP
48 hr
Cell viability (OD570 nm)
1.2
0.9
0.6
0.3
0.0
Control
FN
15.1
30.2
75.5
FN-AgNP
E
24 hr
ROS generation ability (relative fold)
1.2
0.9
0.6
0.3
0.0
Control
FN
15.1
30.2
75.5
FN-AgNP
48 hr
ROS generation ability (relative fold)
1.2
0.9
0.6
0.3
0.0
Control
FN
15.1
30.2
75.5
FN-AgNP

Figure S1. Cytoskeletal fibers examination and Migration ability of HSF. **(A)** Rhodamine phalloidin staining for the cytoskeletal fibers of HSF in pure FN and different concentrations of FN-AgNP nanocomposites at 48 hr observed by fluorescence microscopy. F-actin: red color fluorescence, DAPI: blue color fluorescence. Scale bar = 50 μm. **(B)** HSF cell migration into gap zone area was monitored by fluorescence microscopy. After incubating for 24 and 48 hours, the cells were stained by calcein-AM (2 μM) prior to the examination. Scale bar = 200 μm. **(C)** The length of F-actin fiber was detected for HSF on various materials and the results were semi-quantified at 48 hours. **(D)** The migration distance of HSF seeding on different materials was semi-quantified at 48 hours. Data are the mean ± SD (n = 3). *p<0.05; **p<0.01: greater than the control. **(E)** HSF proliferation examined by MTT assay on pure FN and FN-AgNP nanocomposites containing ~15.1 ppm, ~30.2 ppm and ~75.5 ppm of AgNP. Data are the mean ± SD (n = 6). *p<0.05; **p<0.01: greater than the control. All the results are representative of one of six independent experiments. The intracellular ROS was quantified by 2,7- dichlorofluorescein diacetate (DCFH-dA) and flow cytometric analysis for **(F)** HSF on different materials. Data are mean ± SD (n = 3). *p<0.05; **p<0.01: smaller than the control. All the results are representative of one of three independent experiments.

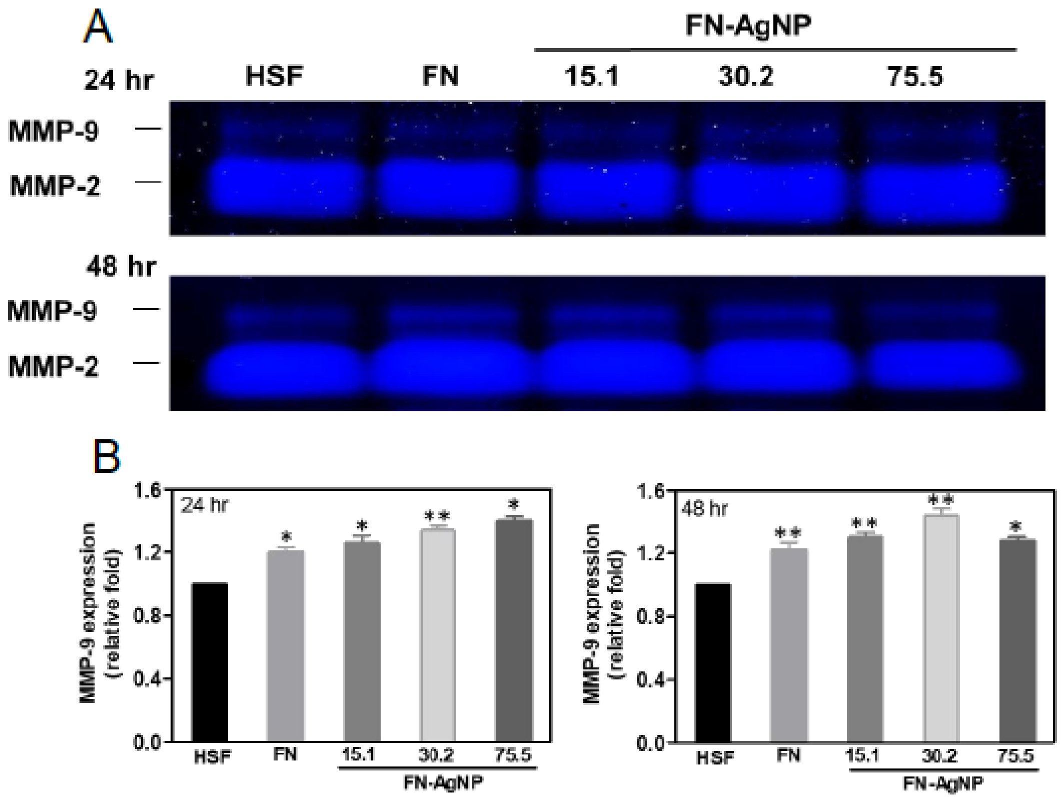

Figure S2. The MMP enzymatic activities and expression of HSF culturing on pure FN and different

concentration of FN-AgNP at 24 and 48 hours. **(A)** A representative zymogram for MMP-2 and MMP-9 at 24 and 48 hours is shown. **(B)** The semi-quantitative measurement of the optical density (OD) of gelatinolytic bands indicated significantly higher MMP-9 expression for HSF on FN-AgNP 30.2 ppm at 24 and 48 hours. Data are the mean ± SD (n = 3). *p<0.05; **p<0.01: greater than control (HSF)

Figure S3

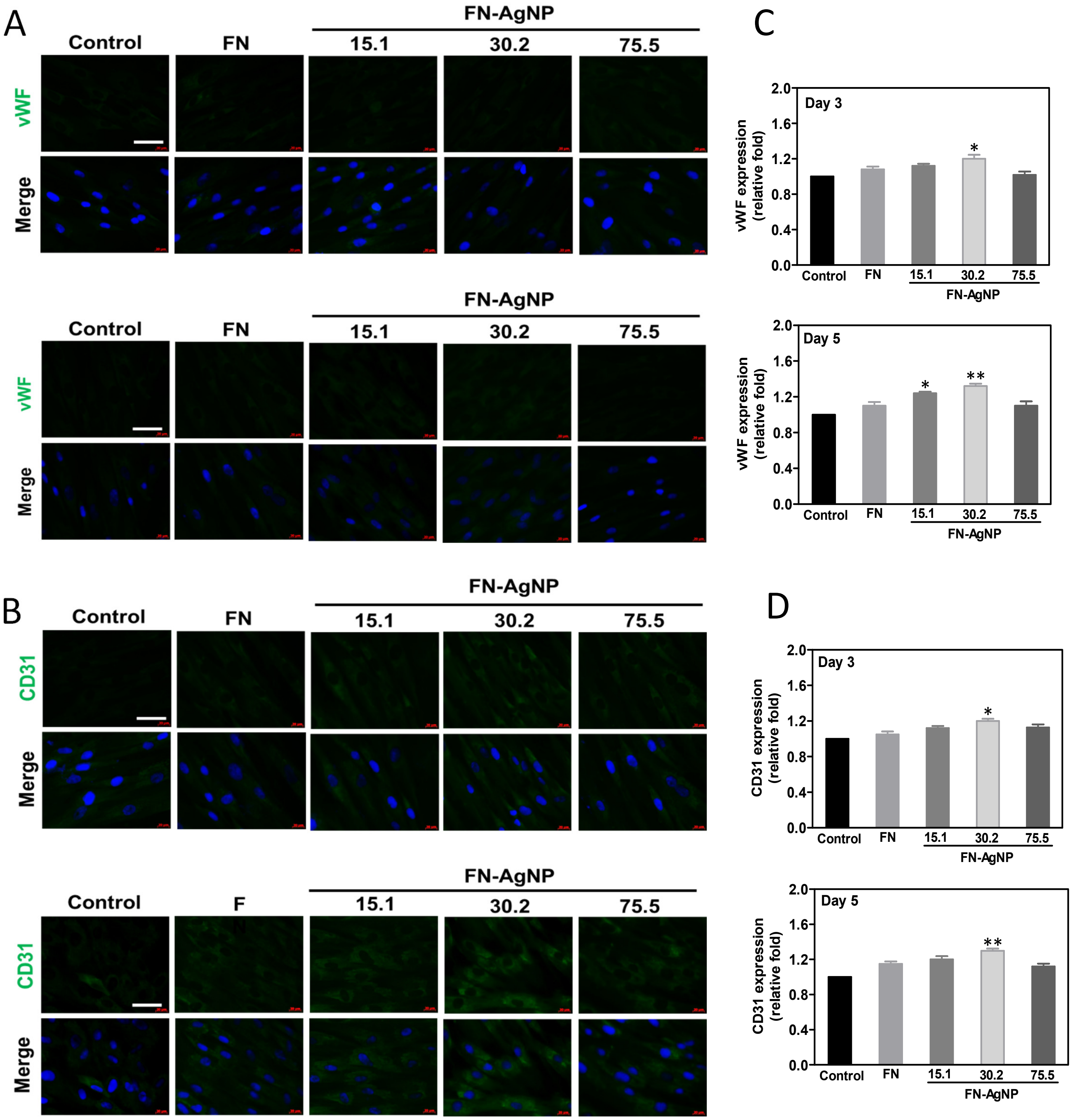

Figure S3. Differential expression of vWF and CD31 and protein in MSCs culturing with various nanomaterials at days 3 and 5. The MSCs were firstly stained by primary (A) anti –vWF and (B) anti –CD31 antibody and conjugated with FITC secondary antibody (green color). The cell nucleus was located by DAPI (blue color). The images were taken by fluorescence microscopy. Scale bar = 20 μm. (C-D) The quantitative results of fluorescence intensity for vWF and CD31 revealed a higher expression amount in FN-AgNP 30.2 ppm group. Data are the mean ± SD (n = 3). $^*p<0.05$; $^{**}p<0.01$: greater than the control.
